# Study of Nonlinear Optical Properties of a Self-Healing Organic Crystal

**DOI:** 10.1021/acsomega.4c06466

**Published:** 2024-08-26

**Authors:** Clodoaldo Valverde, Francisco A. P. Osório

**Affiliations:** † Laboratório de Modelagem Molecular Aplicada e Simulação (LaMMAS), Campus de Ciências Exatas e Tecnológicas, Universidade Estadual de Goiás, 75001-970 Anápolis, Goiás, Brazil; ‡ Universidade Paulista − UNIP, 74845-090 Goiânia, Goiás, Brazil; § Instituto de Física, Universidade Federal de Goiás, 74690-900 Goiânia, Goiás, Brazil

## Abstract

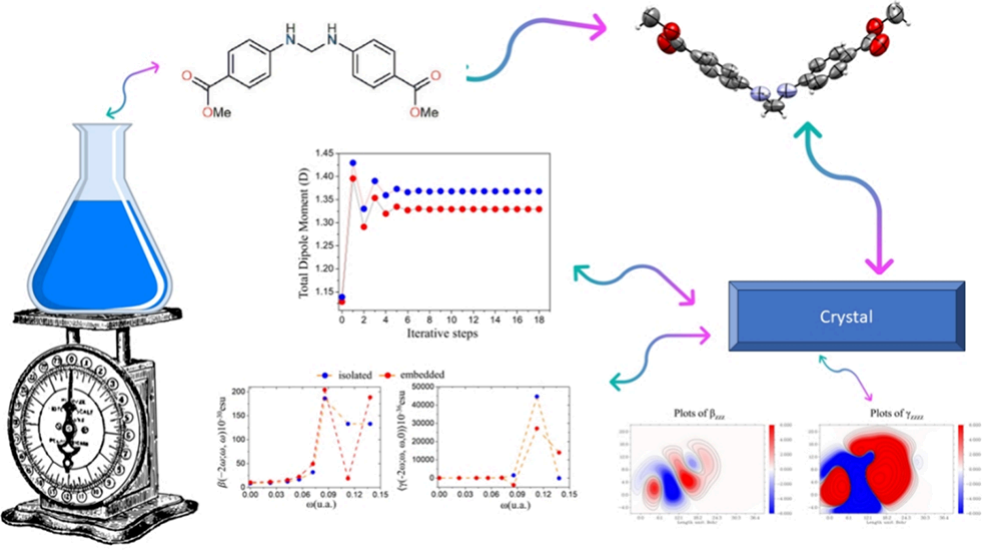

Recently a noncentrosymmetric single crystal of a dibenzoate derivative, namely, dimethyl-4,4′-(methylenebis(azanediyl))dibenzoate, with second harmonic generation activities at 405 nm and ultrafast self-healing activity was reported by Mondal et al. in *Nature Communications* in 2023. Here, the linear and nonlinear optical properties of this notable molecular crystal were simulated using 1,611,464 atoms in the Supermolecule approach at the DFT/CAM-B3LYP/aug-cc-pVTZ level. Our results for the second order nonlinear optical properties of dimethyl-4,4′-(methylenebis(azanediyl))dibenzoate show that the second harmonic generation is more significant at 532 nm. In addition, the density functional theory calculations of the electro-optical parameters for the crystals in the pristine state and after the fracture mechanical self-healing process show small differences, confirming the efficiency of the self-healing process. Additionally, the crystal displays significant third-order nonlinear optical properties, particularly pronounced at a shorter wavelength of 330 nm. Thus, the self-healing dimethyl-4,4′-(methylenebis(azanediyl))dibenzoate crystal shows relevant second and third order nonlinear optical properties which make it a very interesting material for optical applications.

## 1 Introduction

The study of the nonlinear optical properties of synthetic crystals has been a fascinating and promising field of research, with diverse applications including piezoelectric transducers,^1−4^ energy storage,^3−5^ and nonlinear optical materials.^6−9^ An intriguing and highly desirable property in crystalline materials is the ability for self-healing,^10−12^ a characteristic that significantly enhances the durability and efficacy of materials in various technological applications.^13−15^

Recently, the discovery of noncentrosymmetric organic crystal dimethyl-4,4′-(methylenebis(azanediyl))dibenzoate (DMD) capable of autonomous self-healing within milliseconds was reported by Mondal et al.^16^ This crystal exhibits a remarkable combination of mechanical and optical properties, including the following: (a) ultrafast mechanical actuation, making them promising for use in actuator devices; (b) undergoing mechanical deformation on a millisecond time scale, showing exceptional self-healing, meaning the crystals can autonomously repair themselves after fracturing without the need for external stimuli; this property makes them promising for applications in durable and reliable materials; and (c) nonlinear optical response, meaning the crystals show second harmonic generation (SHG) activity, which is not affected by the self-healing, making them suitable for use in nonlinear optical devices. Mechanical fracture experiments on the crystals revealed that they have a load limit, above which self-healing is no longer effective.

The aim of this work is to study the nonlinear optical properties of the self-regenerative crystals developed by Mondal et al.,^16^ and for this purpose the Supermolecule approach^17,18^ (SM) is used to simulate the crystalline environment for the DMD crystal (pristine and healed). To elucidate the second- and third-order nonlinear optical (NLO) properties of the DMD crystal, computational calculations will be employed at the DFT/CAM-B3LYP/aug-cc-pVTZ level, a combination that offers precision in the analysis of the long-range electrostatic effects. The critical molecular parameters such as the dipole moment, the average linear polarizability, and the first and second order hyperpolarizabilities, as the related susceptibilities for the isolated and embedded molecules of DMD, as a function of the applied electric field frequencies were calculated, and the results analyzed. We hope that this study can contribute to the understanding and application of crystals with mechanical self-healing properties in the field of nonlinear optics.

## 2 Materials and Methods

### 2.1 Crystal Structures of DMD, Pristine and Self-Healed

**Figure 1:**
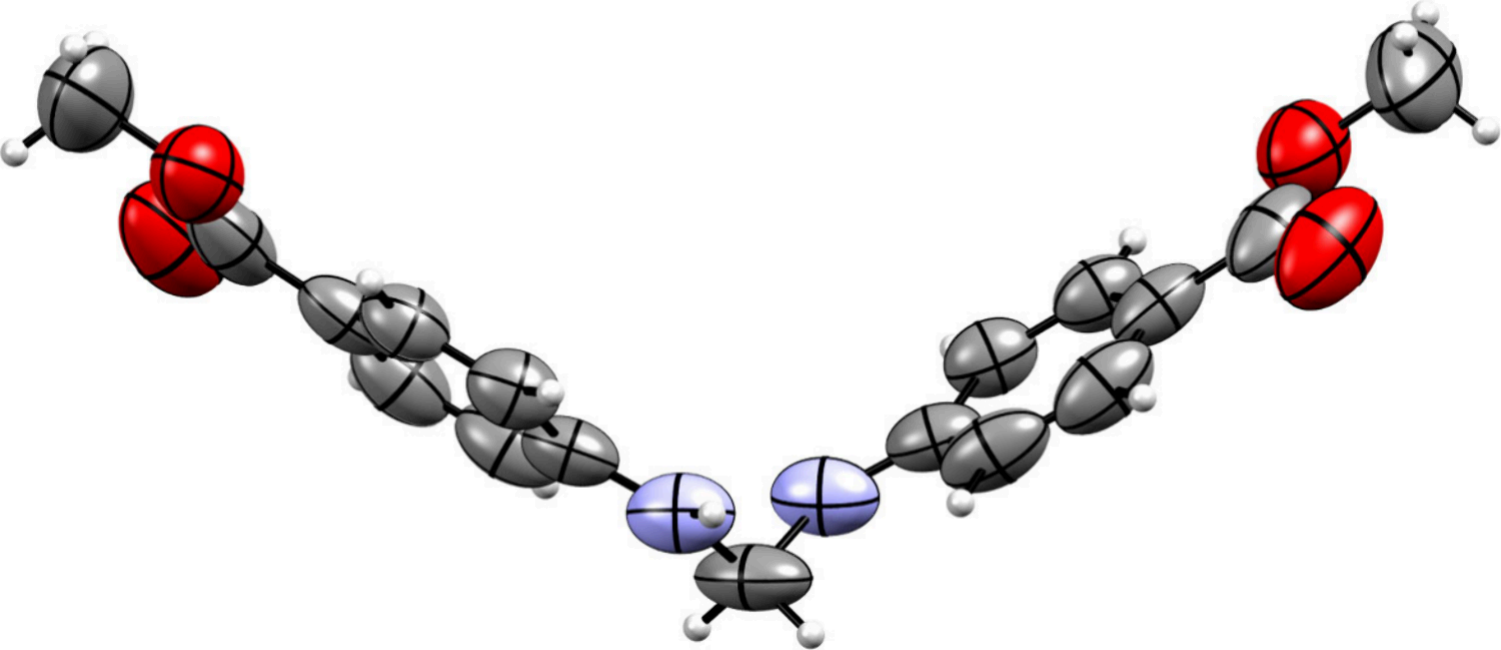
DMD isolated molecule.

The dimethyl-4,4′-(methylenebis(azanediyl))dibenzoate (DMD), with formula C_17_H_18_N_2_O_4_, crystallizes in the tetragonal noncentrosymmetric polar space group *I*4_1_*cd* with half molecule in the asymmetric unit, and eight molecules (Z = 8) per unit cell. The lattice parameters for the pristine crystal are *a* [Å] = 19.25900(10), *c* [Å] = 8.69590(10) with the unit cell volume *V* = 3225.39(5) Å^3^, and the values for the healed crystal are *a* [Å] = 19.25630(10), *c* [Å] = 8.69990(10) with the unit cell volume *V* = 3225.97(5) Å^3^, and as it can be seen after the self-restoring process the structural properties of the crystal have practically not presented significative change. Due to the presence of an sp3-hybridized, tetrahedral methylene connector (−CH2−), the DMD molecule adopts a V-shaped geometry (Figure 1), with an angle of 105.48° between the two arms, as reported by Mondal et al.^16^

The DMD isolated molecule displays a distinct V-shaped geometry due to the sp3-hybridized, tetrahedral methylene connector.

### 2.2 Crystalline Environment Simulation

**Figure 2:**
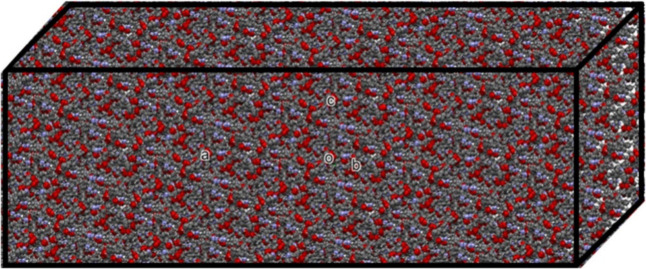
Bulk of the DMD structure.

The Supermolecule (SM) approach is a computational technique utilized for estimating the electrical properties of a crystal, considering the polarization effect. The numerous references to the SM method in research studies^18−23^ attest to its widespread adoption. This approach is grounded in the predominance of intermolecular electrostatic interactions and integrates long-range electrostatic effects. It operates based on the experimental geometry of the crystal’s asymmetric unit. The assembly of the crystal is established by replicating the unit cell in a configuration of 17 × 17 × 17 unit cells; see Figure 2, resulting in a total of 39,304 molecules. Each molecule comprises 41 atoms, amounting to a total of 1,611,464 atoms with all atoms surrounding the asymmetric unit (isolated molecule) considered as point charges.

**Figure 3:**
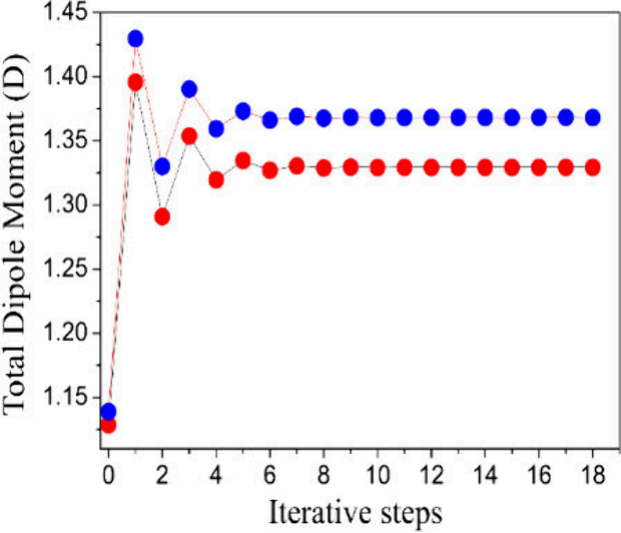
Total dipole moment as a function of iterative steps, for both the pristine DMDp (red) and self-healed DMDh (blue) bulk.

The iterative process of the SM approach begins with the charge’s analysis of an isolated molecule. At this stage, we employ the ChelpG method to calculate the atomic charges of each atom in the isolated molecule as well as other properties, such as the total dipole moment, average linear polarizability, and first and second order hyperpolarizabilities. Subsequently, within the unit cells that compose the crystal, each atom of the molecules is replaced by its partial atomic charge, which was obtained in the previous step. This procedure is repeated multiple times: at each stage, the partial atomic charges are updated and the initial calculations are redone. This cycle continues until the total dipole moment stabilizes, indicating that the electrical properties of the molecule have reached a stationary state, as illustrated in Figure 3. The bulk of the simulated crystal was built for both the pristine (DMDp) and self-healed (DMDh) molecules, and as can be seen from Figure 3, the converged electric dipole moments, 1.33 D and 1.37 D, respectively, show a small variation of ∼3%.

The iterative refinement of charges in the SM approach progressively stabilizes the dipole moments of the molecules, highlighting the robustness of this computational technique in modeling electrical properties.

### 2.3 Autocure Properties and Mechanical Resilience of DMD Crystals: Insights from Recent Research

Recent studies by Mondal et al.^16^ have provided substantial insights into the autocure capabilities of DMD crystals, which demonstrate the potential to rapidly restore their structural and optical properties postmechanical damage. These crystals exhibit an intrinsic ability to self-repair without external stimuli, a process that occurs within milliseconds, driven by electrostatic forces generated at the fracture surfaces. The molecular structure of DMD, featuring flexible bonds and functional groups, significantly enhances the efficiency of this autocure process. Further, the observed mechanical properties make DMD crystals particularly suitable for nonlinear optical applications where the structural integrity impacts the crystal performance. The findings of Mondal et al.^16^ underscore the practical applications of DMD in technology sectors requiring durable and self-sustaining materials.

### 2.4 Electrooptical Parameters

The dipole moment (μ), the average linear polarizability (⟨α⟩), and the linear refractive index were calculated using the equations
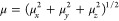
1
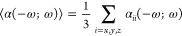
2
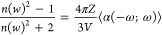
3where *Z* is the number of molecules in the unit cell and *V* is the volume of the unit cell.

The Clausius-Mossotti relation,^24^eq 3, links the average linear polarizability ⟨α(−ω;ω)⟩ to the crystal’s linear refractive index (*n*(ω)).

The magnitude of the total first hyperpolarizability (*β*_*tot*_) is defined by
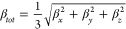
4where
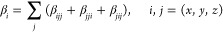
5and the respective second order susceptibility is given by
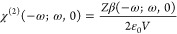
6The average second hyperpolarizability was calculated using the equation
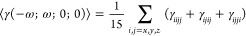
7

Since the optical dispersion was not considered, the static average second hyperpolarizability was obtained using the Kleinmann expression,^25^
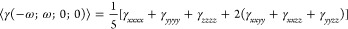
8

The third-order nonlinear susceptibility χ^(3)^ (−ω;ω;ω;−ω), associated with the intensity-dependent refractive index IDRI process, is connected to the average second hyperpolarizability through a specific mathematical relationship,
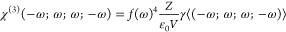
9where  is the Lorentz factor, *Z* is the number of molecules in the unit cell, *V* is the volume of the unit cell, and the IDRI second hyperpolarizability was obtained using the equation,
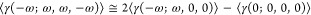
10

All calculations were performed at DFT/CAM-B3LYP/aug-cc-pVTZ level of theory using the Gaussian 16 package^26^ and converted by the electronic units (*esu*).

## 3 Results and Discussion

As highlighted previously, the structural parameters of the DMD crystal before and after the self-healing process practically do not show any significative change. However, other electro-optical parameters show small variations, as for example the total dipole moment that for DMDp (μ_*p*_) is oriented along of the *z*-direction, and for DMDh, *μ⃗*_*h*_, has three components, namely, *μ*_*hx*_ = 0.0630 *D*, *μ*_*hy*_ = 0.0630 *D*, and μ_*hz*_ = 1.36799 *D*, representing an angular deviation of 0.7° in relation with the *z*-axis. Although the magnitude of the Cartesian components, *μ*_*x*_ and *μ*_*y*_, is small, they show the difference between the crystal’s geometry before and after the self-healing process. This fact also can be observed for the static *β*_*i*_-components used to calculate *β*_*tot*_ (eq 5). For DMDp *β*_*tot*_ = *β*_*z*_/3, but for DMDh all the *β*_*i*_-components are *β*_*x*_ = 0.0523 × 10^–30^*esu*, *β*_*y*_ = 0.0963 × 10^–30^*esu*, and *β*_*z*_ = 31.4015 × 10^–30^*esu*. However, the magnitudes of *β*_*tot*_ for DMDp and DMDh are 10.6494 × 10^–30^*esu* and 10.4672 × 10^–30^*esu*, respectively, showing a perceptual difference of only 1.7%. As we can see, the changes in the static second order NLO parameters obtained with the DFT calculations, for DMDp and DMDh, are small, confirming the excellent restoring process of the DMD crystal. Due to these small differences between the parameter’s results obtained for the pristine and healed crystals, we will limit ourselves to presenting the results of our calculations for the pristine crystal.

**Figure 4:**
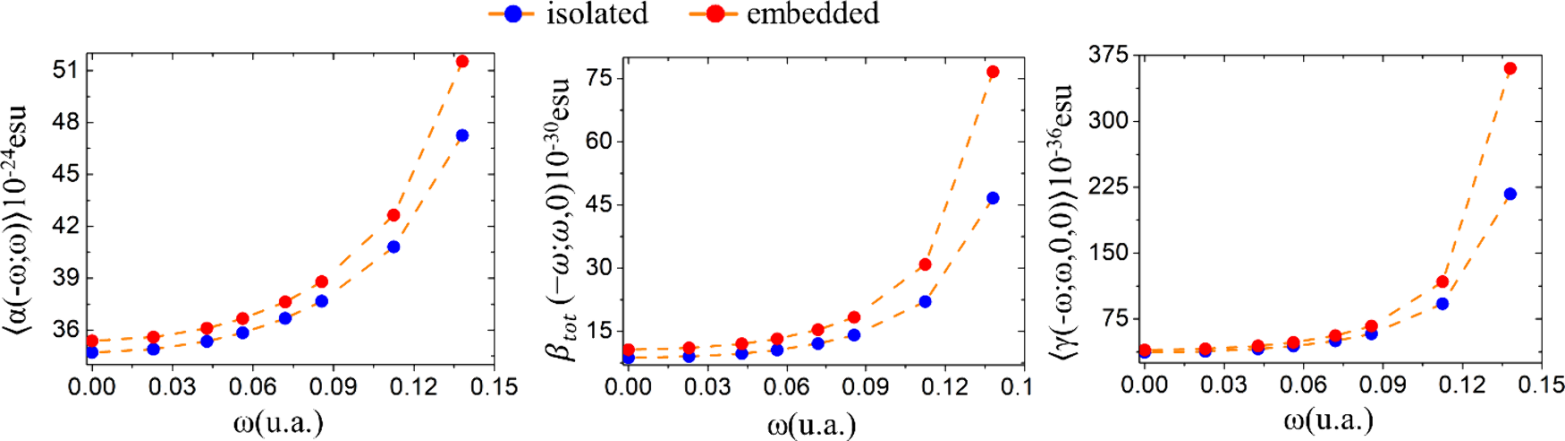
Linear polarizability and the first and second hyperpolarizabilities for DMDp as a function of the electric field frequencies.

The DFT/CAM-B3LYP/aug-cc-pVTZ results for the dynamic electro-optical parameters of the DMDp crystal: ⟨α(−ω;ω)⟩,*β*_*tot*_(−ω;ω,0) and ⟨γ(−ω;ω,0,0)⟩, are shown in Figure 4 for both the isolated and embedded molecules as a function of the several electric field frequencies in the range of 0.0 a.u. ≤ ω ≤ 0.138 a.u. As can be seen, the crystalline environment polarization effects enhance the electro-optical parameter values of ⟨α(−ω;ω)⟩, *β*_*tot*_(−ω;ω,0) and ⟨γ(−ω;ω,0,0)⟩ in the static case (ω = 0.138 a.u.) of 2% (9%), 21.8% (64%), and 7% (66%), respectively. Additionally, Figure 3 shows the dispersion relationship of these parameters with the increasing of the electric field frequency; the qualitative behaviors of the curves are similar, increasing with the increase of the electric field frequency, and enhancement at the parameter-values becomes more abrupt, especially for the embedded molecules, and for frequencies higher than ω = 0.08565 a.u. (λ = 532 nm).

The displayed frequency-dependent behaviors of linear polarizability and hyperpolarizabilities in the DMDp crystal show the significant influence of the crystalline environment on these electro-optical parameters, as they intensify with increasing electric field frequencies.

**Figure 5:**
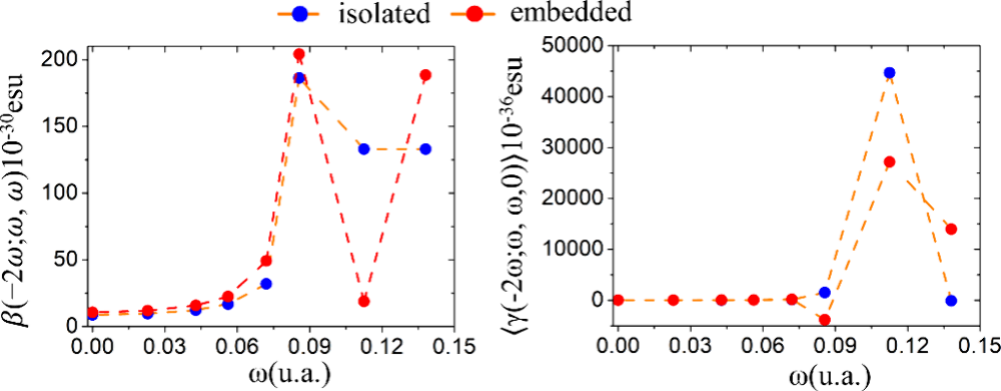
Dispersion relation for the SHG first hyperpolarizabilities and the resonance for wavelengths smaller than 532 nm.

Figure 5 shows the second harmonic generation parameters β(−2ω;ω,ω) and γ(−2ω;ω,ω,0) as a function of the electric field frequencies for isolated and embedded DMDp molecules. As we can see, the parameter-values for frequencies lower than 0.07200 a.u. (632.8 nm) present a smooth increase with the increasing of the electric field frequency, and the nonlinear nature of the dispersion relationships appears in Figure 4, at frequency range 0.085 a.u. (532 nm) ≤ ω ≤ 0.0138 a.u. (330 nm).

For analyzing with more detail the second and third order NLO properties in the region of short wavelengths, Table 1 shows the values of β(−2ω;ω,ω) and ⟨γ(−ω;ω,ω,ω)⟩ for DMDp isolated and embedded molecules. The crystalline environment polarization increases the parameter values except the β(−2ω;ω;ω)-value at 405 nm, and at this wavelength the embedded value is 15% of that for the isolated molecule. Using eq 6 the second-order nonlinear susceptibility (χ(^2^) (−2ω;ω,ω)) was calculated, and the obtained values were 106.18, 9.82, and 98.04 pm/V at electric field wavelengths of 532, 405, and 330 nm, respectively. Mondal et al.^16^ have reported the ability of DMD to generate second harmonicity at λ = 405 nm. Our results for the second order susceptibility (χ^(2)^) at 532 nm suggest that the maximum intensity of SGH can occur if the sample was excited at 1064 nm.

**Table 1:** *β*_*tot*_ (−2ω;ω,ω)(10^–30^*esu*) and ⟨γ(-ω;ω,ω,ω)⟩(10^–36^*esu*) for Three Values of the Electric Field Wavelength

	*β*_*tot*_(−2ω;ω,ω)		⟨γ(−ω;ω,ω,ω)⟩	
λ (nm)	Isolated	Embedded	Isolated	Embedded
532	186.30	204.16	79.28	96.78
405	133.04	18.88	147.50	197.81
330	132.99	188.51	397.39	683.52

Table 1 also shows the IDRI second hyperpolarizability values, ⟨γ(−ω;ω,ω,ω)⟩, at 532, 405, and 330 nm, and as can be seen the values increases with the increasing of the electric field frequencies; the γ-value at 330 nm is more than three times the value at 405 nm and almost seven times the value at 532 nm. The UV absorption spectrum presents a prominent peak at 260 nm, far from 330 nm, the short wavelength limit used in the NLO calculations.

### 3.1 Contributions of Atomic Components to the First Hyperpolarizability

To better understand the contributions of each atom in the DMD isolated molecule to the hyperpolarizability, the hyperpolarizability density analysis method^27−30^ was used, which effectively identifies how different regions within a molecule contribute to its hyperpolarizability. The electronic density of a system, represented as ρ(*r⃗*,*F⃗*), is expanded using a Taylor series relative to the external electric field *F⃗*. This detailed approach sheds light on the influence of specific molecular sections on total hyperpolarizability, which is crucial for exploring nonlinear optical properties.

The electronic density, ρ(*r⃗,F⃗*) in relation to the applied electric field *F⃗*, can be expanded as follows:
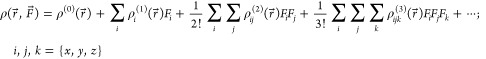
11

Based on the previously mentioned equation and the expansion of the dipole moment in powers of *F⃗*, the components of the first and second hyperpolarizabilities can be formulated as follows:
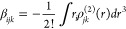
12and
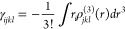
13

In this study, we focus solely on the most significant component, *β*_*zzz*_, of the static first hyperpolarizability tensors, which is determined using the formula below:
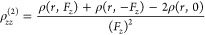
14and
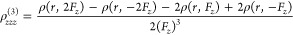
15where
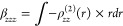
16where
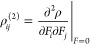
17and
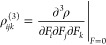
18

In this scenario, ρ(*r⃗*,*F*_*Z*_) denotes the electron density at a specific point *r⃗* influenced by a low-intensity electric field *F*_*Z*_. The aug-cc-pVTZ basis set was utilized to calculate this parameter. A finite difference step size of 0.003 atomic units (a.u.) was chosen for *F*_*Z*_, which is considered as adequate for precise calculations.^27^ Evidence supporting this affirmation is shown in the last two rows of Table 2, where the values of β_*zzz*_ and *γ*_*zzzz*_, derived from integrating the densities of the first and second hyperpolarizabilities at *F*_*Z*_ = 0.003 a.u., align well with those determined through the Coupled-Perturbed Kohn–Sham (CPKS) method.

**Table 2:** Component of First Hyperpolarizability β_*zzz*_ and Second Hyperpolarizability *γ*_*zzzz*_ (a.u.) for Different Atoms in the Isolated DMD Molecule

(I)		
Atoms	β_*zzz*_ (a.u)	*γ*_*zzzz*_ (a.u.)
O	33.73	–564.53
N	146.44	2265.11
H	26.44	1036.31
C	–48.71	160.98
C	82.68	1278.12
O	–44.51	–271.47
C	32.91	–574.76
H	11.06	–14.57
C	45.66	1546.38
H	22.98	31.07
C	–91.75	–1090.58
C	–39.63	3934.03
H	–29.70	1624.61
C	–26.00	331.46
H	15.94	126.37
C	–13.86	2339.87
H	–113.38	7958.31
C	7.18	15.59
H	6.71	89.07
H	6.14	119.84
H	8.26	–84.03
O	33.60	–564.41
N	146.37	2265.40
H	26.44	1036.33
C	–48.69	160.76
C	82.68	1278.13
O	–44.50	–271.37
C	32.93	–574.70
H	11.06	–14.55
C	45.71	1546.88
H	22.98	31.09
C	–91.78	–1090.98
C	–39.36	3929.67
H	–29.70	1624.50
C	–25.98	331.00
H	15.94	126.35
H	–113.38	7958.35
C	7.16	15.37
H	6.71	89.06
H	6.14	119.86
H	8.26	–84.02
	∑*β*_*zzz*_ = 91.19	∑*γ*_*zzzz*_ = 38,169.89
	|β_*zzz*_^*CPKS*^| = 83.05	γ_*zzzz*_^*CPKS*^ = 38,685.40
	Δ% = 9.8	Δ% = 1.3

Table 2 lists the values of the first and second hyperpolarizabilities in the *zzz*-direction, expressed in atomic units, for various atoms within the DMD structure.

Table 2 presents the components of first and second hyperpolarizabilities, β_*zzz*_ and *γ*_*zzzz*_, for different atoms in the isolated DMD molecule, measured in atomic units (a.u.). The table highlights variations in these properties across various atomic types within the molecule. Notably, nitrogen (N) and the last hydrogen (H) listed show exceptionally high values for *γ*_*zzzz*_, indicating significant contributions to the molecule’s overall second hyperpolarizability. Conversely, some carbon (C) atoms display negative β_*zzz*_ values, suggesting their opposing influence on the molecule’s first hyperpolarizability.

The summation of β_*zzz*_ across all atoms yields a value of 91.19 a.u., compared to 83.05 a.u. obtained by the Coupled-Perturbed Kohn–Sham (CPKS) method, reflecting a discrepancy of 9.8%. Similarly, the total *γ*_*zzzz*_ is calculated at 38,169.89 a.u., closely aligning with the CPKS value of 38,685.40 a.u., with a minimal difference of 1.3%. These comparisons underscore the relative accuracy of the hyperpolarizability assessments and highlight the nuanced contributions of individual atomic components to the overall nonlinear optical properties of the molecule.

**Figure 6:**
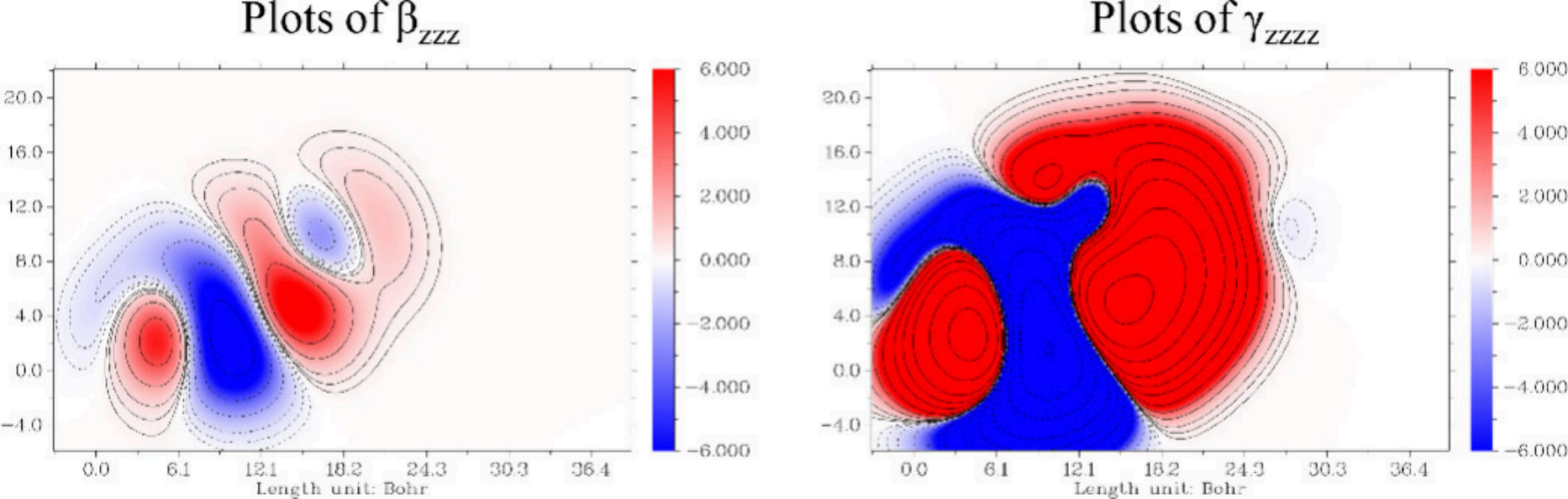
Contour plots of β_*zzz*_ and *γ*_*zzzz*_ densities on the DMD.

The contour plots depicted in Figure 6 illustrate the distribution of the first and second hyperpolarizabilities, β_*zzz*_ and *γ*_*zzzz*_, respectively, within the DMD molecule. The left panel shows the β_*zzz*_ values where the red and blue regions indicate positive and negative contributions. Similarly, the right panel maps the *γ*_*zzzz*_ values with the same color coding. The contours represent different levels of hyperpolarizability intensity with denser regions suggesting stronger effects.

The presence of distinct red and blue areas in both plots indicates areas within the molecule where electron density contributes positively or negatively to the overall hyperpolarizability. Such detailed visualization is crucial for understanding how different molecular segments contribute to the nonlinear optical properties of the molecule. Notably, regions with intense red suggest areas with strong positive hyperpolarizability, which are essential for applications that rely on enhanced nonlinear optical responses.

### 3.2 Assessment of Third-Order Nonlinear Susceptibility and Refractive Index in the DMDp Embedded Molecule

The values of the third order nonlinear susceptibility, χ^(3)^(−ω;ω,ω,−ω), for DMDp embedded molecules, are shown in Table 3. As can be seen, the χ^(3)^-values are large enough to characterize the DMDp crystal as an optical material, with interesting and promising third-order optical properties. The result at 532 nm,  is an expressive value, practically equal to the experimental value measure by Z-scan for the nonlinear optical material 3MPNP, .^31^ On the other hand, the χ^(3)^-values at 405 and 330 nm are 2.57 and 18.3 times this experimental value.

**Table 3:** Third Order Macroscopic Susceptibilities for the DMDp Crystal

λ (nm)	*n*(ω)	χ^(3)^(−ω;ω;ω;−ω) × 10^–20^ *esu*
∞	1.66	0.91
1906.4	1.66	0.99
1064	1.67	1.17
810	1.69	1.41
632.8	1.71	1.89
532	1.74	2.64
405	1.84	7.12
330	2.11	50.77

The refractive index data from Table 3 provide key insights into the optical properties of the DMD crystal. The refractive index, *n*(ω), is a critical parameter indicating how much the material slows light passing through it, directly affecting the efficiency of nonlinear optical properties such as third-order generation, χ^(3)^.

The table demonstrates a variation in the refractive index across different wavelengths, increasing from 1.66 to 2.11 as the wavelength decreases from infinity to 330 nm. This trend suggests a direct relationship between decreasing wavelength and increasing refractive index. This variation implies normal optical dispersion, where the phase velocity of light decreases at shorter wavelengths. The increase in the refractive index at shorter wavelengths suggests enhanced light–matter interactions at these wavelengths. This is supported by the rising values of χ^(3)^, peaking at 50.77 × 10^–20^ esu at 330 nm, facilitating stronger nonlinear phenomena and making the material more efficient for applications requiring significant nonlinear susceptibilities, such as frequency generation or advanced optical processing.

Table 4 compares the third-order nonlinear optical susceptibility (χ^(3)^(−ω;ω,ω,−ω)) of the DMDp crystal to other organic nonlinear crystals.

**Table 4:** Third-Order Nonlinear Optical Susceptibility for DMDp Crystals Compared with the Dynamic Experimental Results for Some Organic Nonlinear Crystals

	λ (nm)	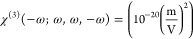
DMDp (present work)	532	2.64
(2E)-3-(3-methylphenyl)-1-(4-nitrophenyl)prop-2-en-1-one (3MPNP)^31^	532	2.771
1-(5-chlorothiophen-2-yl)-3-(2,3-dimethoxyphenyl)prop-2-en-1-one (CTDMP)^31,32^	532	0.2383
(2E)-3[4(methylsulfanyl)phenyl]-1-(4-nitrophenyl)prop-2-en-1- one (4N4MSP)^31,32^	800	0.0237
(2E)-1-(4-bromophenyl)-3-[4(methylsulfanyl)phenyl]prop-2-en-1-one (4Br4MSP)^31,33^	800	0.0230
(2E)-1-(3-bromophenyl)-3-[4(methylsulfanyl)phenyl]prop-2-en-1-one (3Br4MSP)^31,32^	800	0.0199

The DMDp crystal exhibits significant third-order nonlinear optical susceptibility (χ^(3)^) at 532 nm, comparable to 3MPNP and much higher than those of other analyzed compounds, especially at longer wavelengths. These results highlight the potential of DMDp for applications in nonlinear optical devices operating at shorter wavelengths.

Moreover, the observed variation in the refractive index, particularly the notable increase at shorter wavelengths, is advantageous for designing photonic devices such as waveguides or optical resonators. Precise control of the refractive index is crucial for optimizing light confinement and propagation, making DMDp a promising material for these applications.

## 4 Conclusion

In conclusion, in the present work, a detailed study of the second- and third-order nonlinear optical properties of the noncentrosymmetric molecular crystal, dimethyl-4,4′-(methylenebis(azanediyl))dibenzoate (DMD)) with self-healing activity were reported. Using the Supermolecule approach, the crystalline environments were simulated for the pristine as well as for the self-healed DMD samples. At the DFT/CAM-B3LYP/aug-cc-pVTZ level we have calculated the electro-optical parameters of the DMD crystal, before and after the self-healing; our theoretical results at the static regime confirm that the difference between the NLO parameters of crystals is negligible.

The electric dipole moment and the static first hyperpolarizability for the sample after the self-healing process present perceptual increases of 2.9% and 1.7%, respectively, as compared with the results for the pristine sample, confirming that the self-healing process has preserved the physical and structural properties of the DMD samples. The results of the electrooptical parameters for the pristine DMD showed important nonlinear effects in the region of small wavelengths; particularly, the second-order nonlinear susceptibility at 532 nm, χ^2^ = 106.18 pm/V, is a significant value and shows the excellent second-order nonlinear property of DMD.

Furthermore, the third-order nonlinear susceptibility does present significant values in all frequency ranges, and in the study here, the values varied between 0.90 and 50.7 × 10^–20^ (m/V)^2^. Therefore, the DMD is a potential material to be studied as an optical material due to its nonlinear optical properties in the small wavelength region and its mechanical self-healing properties.
